# Bibliometric comparison of Nobel Prize laureates in physiology or medicine and chemistry

**DOI:** 10.1007/s00210-024-03081-z

**Published:** 2024-04-23

**Authors:** Severin Bünemann, Roland Seifert

**Affiliations:** Institute of Pharmacology, Hannover Medical School, Carl-Neuberg-Straße 1, 30625 Hannover, Germany

**Keywords:** Nobel Prize, Bibliometric comparison, Gender research, H-index, Citations

## Abstract

The Nobel Prize is an annual honor awarded to the researchers who have made the greatest contribution to humanity with their work in the year in question. Nobel Prizes for physiology or medicine and chemistry most often have direct or indirect pharmacological relevance. In this study, we performed a bibliometric analysis of Nobel Prize laureates from 2006 to 2022. The parameters include the nationalities and age of the laureates, age at their productivity peaks, the research locations, the H-index, the age-adjusted H-index, and the number of citations and publications, and, for each parameter, a comparison of female and male award laureates. Men were much more often awarded the Nobel Prize than women. Surprisingly, women were younger than their male colleagues at the time of the award although the productivity peak was similar. There was a correlation between all publications and the H-index, which was slightly stronger for women than for men. The age-adjusted H-index showed no difference among genders. The USA were the country with the highest number of Nobel Prize laureates, both male and female. Overall, the bibliometric characteristics of male and female Nobel Prize laureates are similar, indicating that among the group of Nobel Prize laureates, there is no bias against women. Rather, the achievements of women are recognized earlier than those of men. The major difference is that the number of women becoming Nobel Prize laureates is much smaller than the number of men. This study provides a starting for future studies with larger populations of scientists to analyze disparities.

## Introduction

The Nobel Prize is an annual award founded by the Swedish engineer, inventor, and entrepreneur Alfred Nobel (1833–1896) (Hansson et al. 2019). The Nobel Prize is awarded to those researchers whose work has been of the greatest benefit to humanity in the year in question. It is awarded in the fields of physics, chemistry, physiology or medicine, literature and peace efforts and is regarded as the highest scientific honor in the respective disciplines. There has also been an award in the field of economics since 1969, but this is not officially categorized as a Nobel Prize.

Since the foundation was established in 1901, 609 Nobel Prizes have been awarded to 975 laureates, of which the Nobel Prize in physiology or medicine has been awarded to 225 persons to date. A Nobel Prize can be awarded to several researchers, each of whom is then considered a Nobel Prize laureate. As a rule, however, a Nobel Prize is not awarded to more than three researchers. The Nobel Prize in physiology or medicine has been awarded by the Nobel Assembly at Karolinska Institute since 1901 (https://www.nobelprize.org/about/the-nobel-assembly-at-karolinska-institutet/; last accessed on 03/18/2024).

In his will, Nobel had stipulated that the prizes should be awarded to the most worthy, regardless of their nationality, and he made no mention of gender. He decided to establish a foundation that would award annual prizes to researchers whose discoveries or inventions had contributed to the well-being of humanity in the previous year (Zárate et al. 2015). The gender gap in the number of Nobel Prize candidates and laureates in the fields of physiology or medicine is striking (Hansson and Fangerau 2018). The Nobel Prize Committee has been criticized for appearing to ignore the contributions of women in science (Mahmoudi et al. 2019; Silver et al. 2018; Valian 2018; Wade 2002). Many Nobel Prizes have direct or indirect pharmacological relevance (Table 1). This background prompted us to perform a bibliometric analysis of the Nobel Prize laureates in physiology or medicine and chemistry (in this field only topics related to pharmacology) from 2006 to 2022. Most importantly, we wished to answer the question whether there is any bias against women in this group.

**Table 1:** Nobel Prize laureates for the years 2006–2022 with a relation to pharmacology

	Year of the Nobel Prize	Name	Gender	Year of birth	Nationality	Field	Research topic honored by the Nobel Prize	Research location in the year of the NP award	Country
1.	2022	Svante Pääbo	m	1955	Sweden	***Physiology or medicine***	For his discoveries about the genomes of extinct hominins and human evolution	Max Planck Institute, Leipzig	Germany
2.	2022	*Carolyn Ruth Bertozzi*	f	1966	USA	**Chemistry**	For the development of click chemistry and bioorthogonal chemistry	Stanford University Medical School, California	USA
3.	2022	Morten Peter Meldal	m	1954	Denmark	**Chemistry**	For the development of click chemistry and bioorthogonal chemistry	University of Copenhagen	Denmark
4.	2022	Karl Barry Sharpless	m	1941	USA	**Chemistry**	For the development of click chemistry and bioorthogonal chemistry	Scripps Research Institute, La Jolla	USA
5.	2021	David Julius	m	1955	USA	***Physiology or medicine***	For their discoveries of the human receptors for temperature and touch sensation	Scripps Research Institute, La Jolla	USA
6.	2021	Ardem Patapoutian	m	1967	USA	***Physiology or medicine***	For their discoveries of the human receptors for temperature and touch sensation	Scripps Research Institute, La Jolla	USA
7.	2020	Harvey J. Alter	m	1935	USA	***Physiology or medicine***	For her discovery of the hepatitis C virus	U.S. National Institutes of Health, Maryland	USA
8.	2020	Michael Houghton	m	1949	UK	***Physiology or medicine***	For her discovery of the hepatitis C virus	University of Alberta, Canada	Canada
9.	2020	Charles M. Rice	m	1952	USA	***Physiology or medicine***	For her discovery of the hepatitis C virus	Rockefeller University, New York City	USA
10.	2020	*Emmanuelle Charpentier*	f	1968	France	**Chemistry**	For the development of a method for genome editing (CRISPR/Cas method)	Humboldt University, Berlin	Germany
11.	2020	*Jennifer Doudna*	f	1964	USA	**Chemistry**	For the development of a method for genome editing (CRISPR/Cas method)	University of California, Berkeley	USA
12.	2019	William G. Kaelin	m	1957	USA	***Physiology or medicine***	For the discovery of molecular mechanisms of oxygen uptake by cells	Harvard Medical School, Boston	USA
13.	2019	Gregg L. Semenza	m	1956	USA	***Physiology or medicine***	For the discovery of molecular mechanisms of oxygen uptake by cells	Johns Hopkins University in Baltimore, Maryland	USA
14.	2019	Peter J. Ratcliffe	m	1954	UK	***Physiology or medicine***	For the discovery of molecular mechanisms of oxygen uptake by cells	University of Oxford	UK
15.	2018	James P. Allison	m	1948	USA	***Physiology or medicine***	For the discovery of a cancer therapy by inhibiting negative immune regulation	University of Texas	USA
16.	2018	Tasuku Honjo	m	1942	Japan	***Physiology or medicine***	For the discovery of a cancer therapy by inhibiting negative immune regulation	Kyoto University	Japan
17.	2018	George P. Smith	m	1941	USA	**Chemistry**	For the phage display of peptides and antibodies	Retirement—University of Missouri	USA
18.	2018	Gregory P. Winter	m	1951	UK	**Chemistry**	For the phage display of peptides and antibodies	Trinity College, Cambridge	UK
19.	2018	*Frances H. Arnold*	f	1956	USA	**Chemistry**	For the directed evolution of enzymes	California Institute of Technology	USA
20.	2017	Jeffrey C. Hall	m	1945	USA	***Physiology or medicine***	For her discoveries concerning the molecular control mechanisms of the circadian rhythm	Retirement—University of Maine near Bangor, Maine	USA
21.	2017	Michael Rosbash	m	1944	USA	***Physiology or medicine***	For her discoveries concerning the molecular control mechanisms of the circadian rhythm	Harvard University, Boston	USA
22.	2017	Michael W. Young	m	1949	USA	***Physiology or medicine***	For her discoveries concerning the molecular control mechanisms of the circadian rhythm	Rockefeller University, New York City	USA
23.	2016	Yoshinori Ohsumi	m	1945	Japan	***Physiology or medicine***	For his discoveries of the mechanisms of autophagy	Retirement—Tokyo Institute of Technology	Japan
24.	2015	William C. Campbell	m	1930	Ireland	***Physiology or medicine***	For their discoveries concerning a novel therapy for malaria	Drew University, Madiso	USA
25.	2015	Satoshi Ōmura	m	1945	Japan	***Physiology or medicine***	For their discoveries concerning a novel therapy for malaria	Kutasato University, Tokyo	Japan
26.	2015	*Tu Youyou*	f	1930	PR China	***Physiology or medicine***	For their discoveries concerning a novel therapy for malaria	China Academy of Traditional Chinese Medicine, Beijing	PR China
27.	2014	John O'Keefe	m	1939	USA	***Physiology or medicine***	For discoveries of cells that form a positioning system in the brain	University College London	UK
28.	2014	*May-Britt Moser*	f	1963	Norway	***Physiology or medicine***	For discoveries of cells that form a positioning system in the brain	Norwegian University of Science and Technology, Trondheim	Norway
29.	2014	Edvard Moser	m	1962	Norway	***Physiology or medicine***	For discoveries of cells that form a positioning system in the brain	Norwegian University of Science and Technology, Trondheim	Norway
30.	2013	James Rothman	m	1950	USA	***Physiology or medicine***	For the discovery of transport processes in cells	Yale University, New Haven	USA
31.	2013	Randy Schekman	m	1948	USA	***Physiology or medicine***	For the discovery of transport processes in cells	University of California, Berkeley	USA
32.	2013	Thomas Südhof	m	1955	Germany	***Physiology or medicine***	For the discovery of transport processes in cells	Stanford University Medical School, California	USA
33.	2012	John Gurdon	m	1933	UK	***Physiology or medicine***	For the discovery that mature cells can be reprogrammed to become pluripotent stem cells	University of Cambridge	UK
34.	2012	Shin`ya Yamanaka	m	1962	Japan	***Physiology or medicine***	For the discovery that mature cells can be reprogrammed to become pluripotent stem cells	Kyoto University	Japan
35.	2012	Robert Lefkowitz	m	1943	USA	**Chemistry**	For their studies on G protein-coupled receptors	Duke University, North Carolina	USA
36.	2012	Brian Kobilka	m	1955	USA	**Chemistry**	For their studies on G protein-coupled receptors	Stanford University Medical School, California	USA
37.	2011	Bruce Beutler	m	1957	USA	***Physiology or medicine***	For their discoveries on the activation of innate immunity	Scripps Research Institute, La Jolla	USA
38.	2011	Jules Hoffmann	m	1941	Luxembourg	***Physiology or medicine***	For their discoveries on the activation of innate immunity	Retirement—University of Strasbourg	France
39.	2011	Ralph M. Steinman	m	1943–2011	Canada	***Physiology or medicine***	For his discovery of dendritic cells and their role in adaptive immunity	posthumously—Rockefeller University, New York City	USA
40.	2010	Robert Edwards	m	1925–2013	UK	***Physiology or medicine***	For its development of in vitro fertilization	University of Cambridge	UK
41.	2009	*Elizabeth Blackburn*	f	1948	Australia	***Physiology or medicine***	For the discovery of how chromosomes are protected by telomeres and the enzyme telomerase	UCSF, San Francisco	USA
42.	2009	*Carol W. Greider*	f	1961	USA	***Physiology or medicine***	For the discovery of how chromosomes are protected by telomeres and the enzyme telomerase	Johns Hopkins University, Baltimore	USA
43.	2009	Jack Szostak	m	1952	UK	***Physiology or medicine***	For the discovery of how chromosomes are protected by telomeres and the enzyme telomerase	Harvard Medical School, Boston	USA
44.	2009	Venkatraman Ramakrishnan	m	1952	India	**Chemistry**	For the studies on the structure and function of the ribosome	Medical Research Council, Cambridge	UK
45.	2009	Thomas A. Steitz	m	1940–2018	USA	**Chemistry**	For the studies on the structure and function of the ribosome	Yale University, New Haven	USA
46.	2009	*Ada Yonath*	f	1939	Israel	**Chemistry**	For the studies on the structure and function of the ribosome	Weizmann Institute of Science, Rechowot, Israel	Israel
47.	2008	*Francoise Barre-Sinoussi*	f	1947	France	***Physiology or medicine***	For the discovery of the HI virus	Institut Pasteur, Paris	France
48.	2008	Luc Montagnier	m	1932–2022	France	***Physiology or medicine***	For the discovery of the HI virus	Queens College of New York University	USA
49.	2008	Harald zur Hausen	m	1936	Germany	***Physiology or medicine***	For his discovery that human papillomaviruses cause cervical cancer	German Cancer Research Centre, Heidelberg	Germany
50.	2007	Mario Capecchi	m	1937	Italy	***Physiology or medicine***	For groundbreaking discoveries in the field of embryonic stem cells and DNA recombination in mammals	University of Utah	USA
51.	2007	Martin Evans	m	1941	USA	***Physiology or medicine***	For groundbreaking discoveries in the field of embryonic stem cells and DNA recombination in mammals	Cardiff University, Wales	UK
52.	2007	Oliver Smithies	m	1925–2017	UK	***Physiology or medicine***	For groundbreaking discoveries in the field of embryonic stem cells and DNA recombination in mammals	University of North Carolina at Chapel Hill	USA
53.	2006	Andrew Z. Fire	m	1959	USA	***Physiology or medicine***	For their discovery of RNA interference	Stanford University Medical School, California	USA
54.	2006	Craig Mello	m	1960	USA	***Physiology or medicine***	For their discovery of RNA interference	Massachusetts Medical School, Worcester	USA
55.	2006	Roger D. Kornberg	m	1947	USA	**Chemistry**	For his work on the molecular basis of gene transcription in eukaryotic cells	Stanford University Medical School, California	USA

Italics: female Nobel Prize laureates

Bold: chemistry

Bold italics: physiology or medicine

We selected the last 15 years at the beginning of the research to capture contemporary research. In addition to that, the history of the Nobel Prize is also a history of changing processes in science and medicine (Hansson et al. 2019). Therefore, we wanted to analyze the current awarding practice. The 16th year was added because it was being awarded when we collected the data to remain as up-to-date as possible. The focus on recent Nobel Prizes also allows us to perform important comparisons with papers on gender aspects in science encompassing a similar historical period (Zehetbauer et al. 2022; Zöllner and Seifert 2024).

Table 1 provides an overview on the Nobel Prize laureates analyzed. The year of award, name, gender, year of birth, nationality of the laureate, research topic honored by the Nobel Prize, research institution, and country of the institution are provided, all publicly available (https://www.nobelprize.org). Every laureate is identified by a number used throughout this paper. We are not considering so much individual laureates in this paper but rather overarching patterns. Only in occasional cases, we mention a specific laureate to highlight a specific trait.

For an in-depth analysis of individual Nobel Prize laureates, the reader is referred to the excellent work of Hansson et al. (2019). The present paper is meant to provide a general bibliometric analysis of contemporary Nobel Prize laureates in the sense of a meta-analysis to identify overarching patterns and mechanisms underlying awarding of the Nobel Prize.

## Materials and methods

The list of Nobel Prize laureates was compiled via the Nobel Prize website (https://www.nobelprize.org). Nobel laureates (*n* = 55) from the field of physiology or medicine and chemistry (in this field only topics related to pharmacology) were listed according to their age and gender, their nationalities, their publications, citations and research rankings, and subsequently their productivity peaks and their research locations. The inclusion criteria were all Nobel Prize laureates from the years 2006–2022 in the fields of physiology or medicine, supplemented by prize laureates in the field of chemistry who were honored for a research topic related to pharmacology.

For each researcher, a bibliometric analysis was performed using the Clarivate database (https://clarivate.com/products/scientific-and-academic-research/research-analytics-evaluation-and-management-solutions/; last accessed 06/08/2023). The Journal Impact Factor, which is calculated annually by Clarivate Analytics and published in the Journal Citations Reports, is widely used to compare journals. It is now frequently used to assess the quality of journals, although this use is controversial. For this work, publication numbers for each research year of each individual Nobel Prize laureate were retrieved and listed in Clarivate with linear regression. Furthermore, with these data, we analyzed the publication peaks of the Nobel Prize laureates. In addition, the nationalities of the Nobel Prize laureates and their location of research were compiled and analyzed from University websites and the Nobel Prize website.

In a further step, the subsequent statistical data analysis was initially carried out by using the *Statistical Package for the Social Sciences software* (SPSS® Version 25), ANOVA (variance analyses of women and men), and the excel program. We used GraphPad 8 to create the graphs with the statistical software R and the package ggplot2 for the relevant tests for frequency distribution, mean value determination, *T*-tests, *p*-tests, Pearson *r*, and the excel program to display the pie charts to illustrate the percentage differences between women and men. Whenever possible and meaningful, the results of women were compared with the results of men. We calculated cross-tabulations with the Cramer-V value and the significances for the number of Nobel Prize laureates, correlations to show the connections between the publications and citations, one-factorial ANOVA calculations and linear regressions to calculate the correlations when comparing female and male Nobel Prize laureates, and mean value determinations to show the comparison of the female and male results and the respective standard deviations. The results were presented and visualized in different graphics to show the respective totality, the female and the male characteristics.

## Results and discussion

**Fig. 1:**
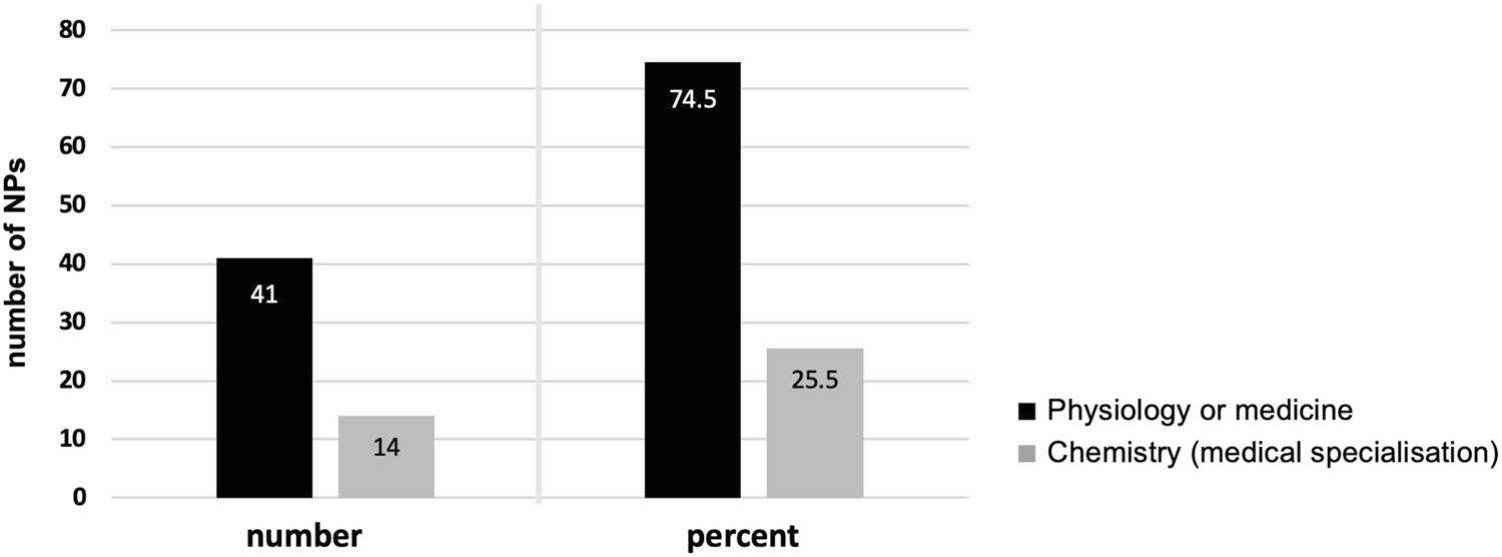
Absolute number and percentage distribution of Nobel Prize laureates. Comparison of the absolute number and percentage distribution of Nobel Prize laureates (2006–2022)

We analyzed 41 Nobel Prize laureates (74.5%) from physiology or medicine, and 14 Nobel Prize laureates (25.5%) from chemistry (Fig. 1).

**Fig. 2:**
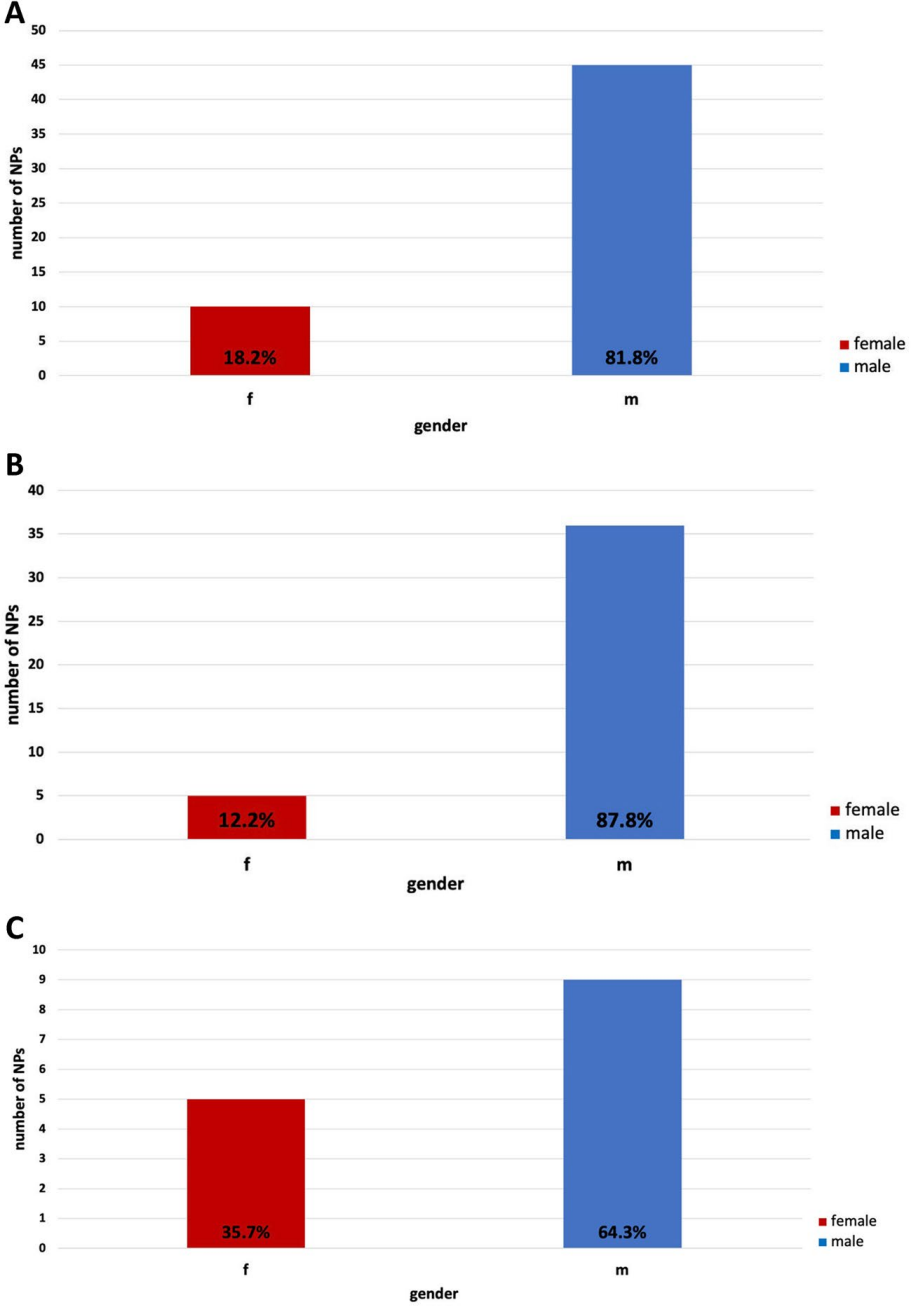
Comparison of the number of Nobel Prize laureates analyzed (Nobel Prize laureates of physiology or medicine and chemistry (in this field only topics related to pharmacology), 2006–2022); **A** overall laureates; **B** NP laureates—physiology or medicine; **C** NP laureates—chemistry

At 18.2%, the proportion of women receiving awards was significantly lower than compared to 81.8% of male award laureates (Fig. 2). There is a clear difference between the genders in the subjects awarded the Nobel Prize: in physiology or medicine, only 14% of the prize laureates were women, while the proportion in chemistry of women was 36%.

**Fig. 3:**
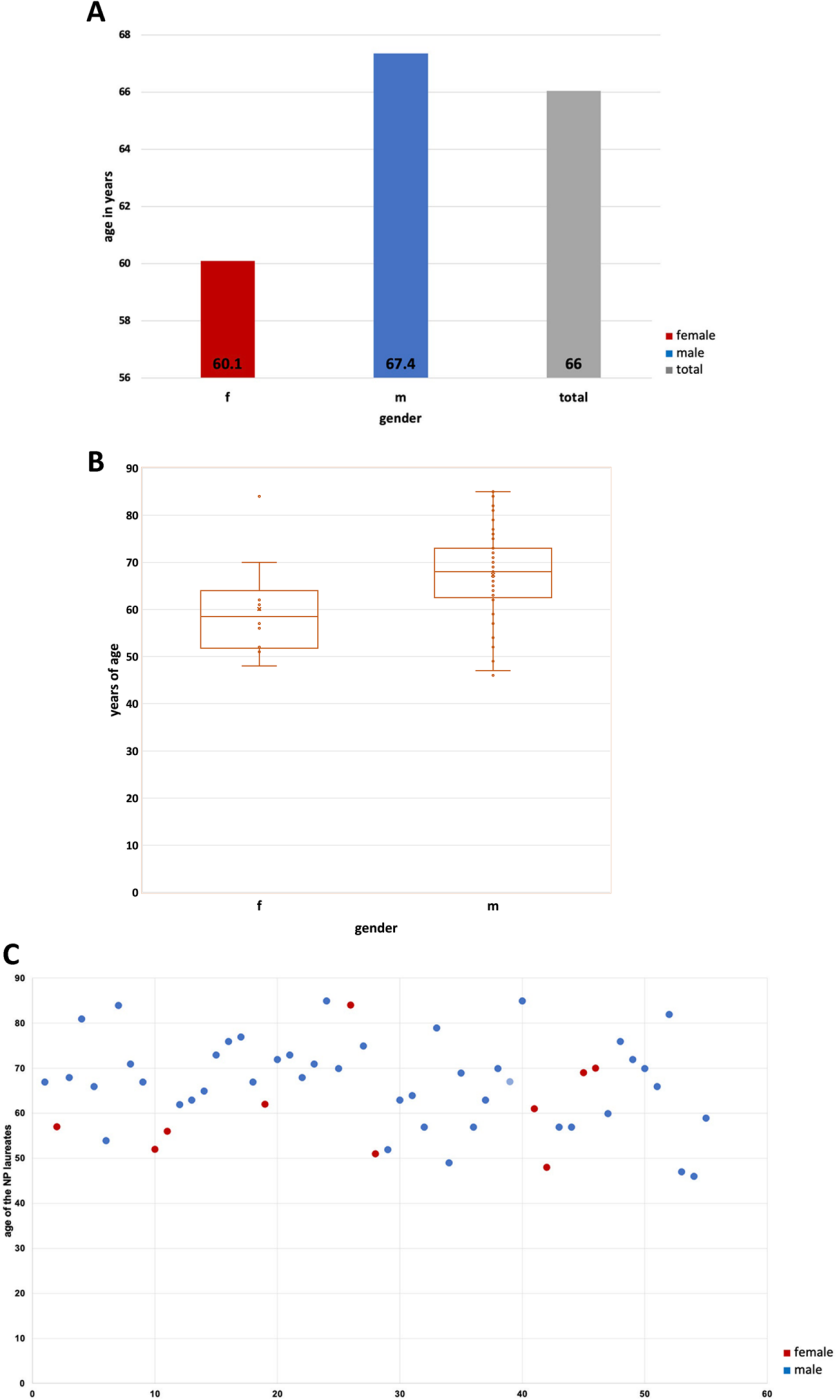
Illustration of the age of Nobel Prize laureates at the time of awarding the Nobel Prize (Nobel Prize lauretaes of physiology or medicine and chemistry (in this field only topics related to pharmacology) 2006–2022); **A** mean values; **B** mean values and SD, difference between genders *p*=0.039 (the *x* is representing the mean value of age: female 60.1 years, male 67.4 years; the o dots are representing the age of each NP laureate; the box corresponds to the area containing the middle 50% of the data; it is bounded by the upper and lower quartiles; the line centered in the box marks the median values); **C** individual laureates; the dots are representing the laureates of Table 1 (in order from 1 to 55; note: one laureate (number 39) was awarded the NP posthumously)

There was a significant difference between the genders (*p* = 0.039) in relation to the average age of Nobel Prize laureates at the time of the Nobel Prize awarding. In average, the age of females was 60.1 years and of males 67.4 years (Fig. 3). The oldest male and female Nobel Prize laureate had an age of 85 and 84 years, respectively. The youngest male and female Nobel Prize laureate had an age of 46 and 48 years, respectively. The standard deviation has a larger range for male Nobel Prize laureates than for female Nobel Prize laureates.

**Fig. 4:**
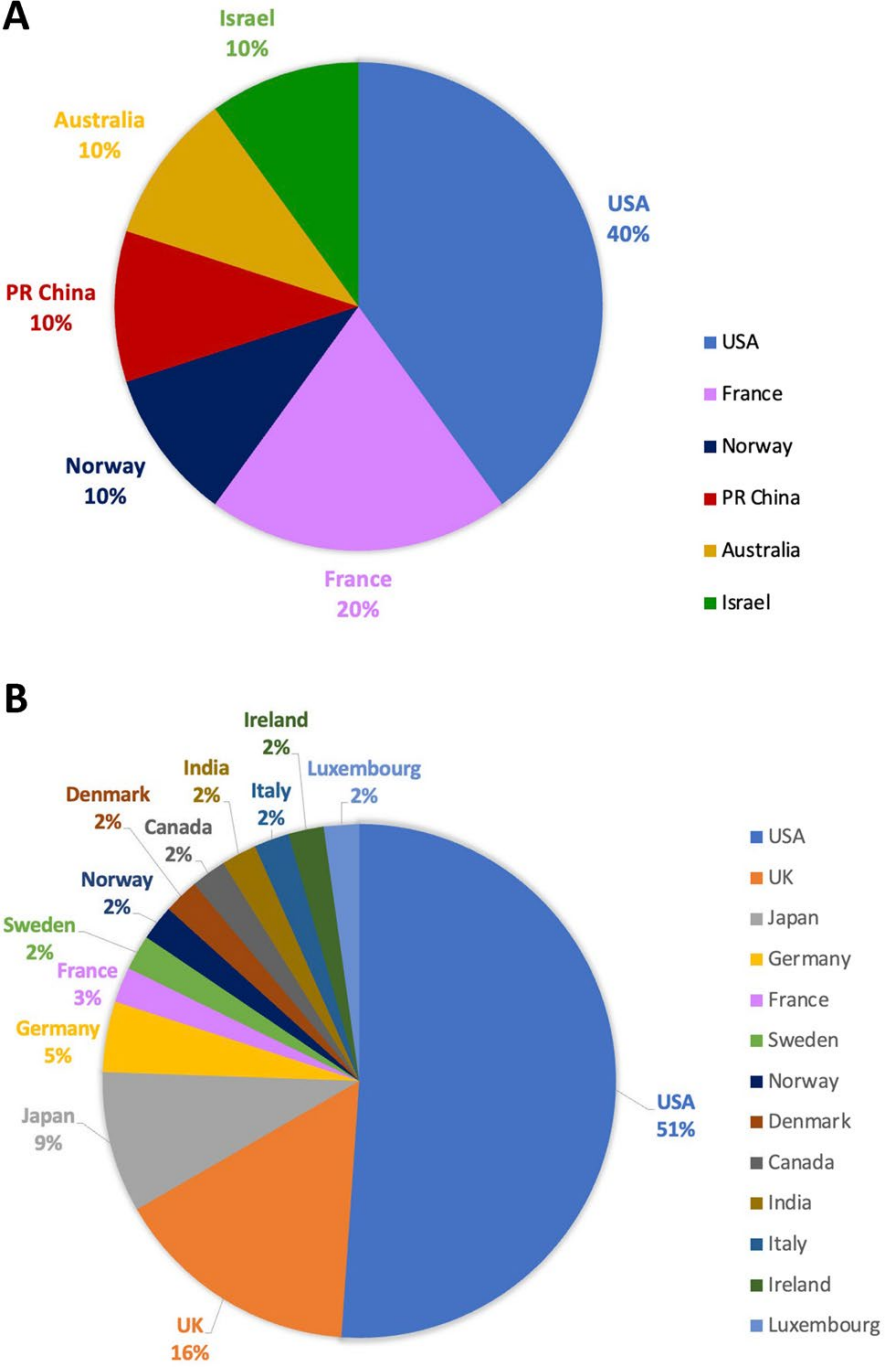
Illustration of the nationalities of the Nobel Prize laureates (2006–2022); **A** female laureates, **B** male laureates

Figure 4 shows the nationalities of the Nobel Prize laureates. The USA dominated Nobel Prize awards, among both women (40%) and men (51%). However, notably, among women, three countries were represented that were not present among men. Specifically, female Nobel Prize laureates were recorded from Israel, Australia, and China. Conversely, the UK, Japan, Germany, Sweden, Denmark, Canada, India, Italy, Ireland, and Luxembourg were represented among men, but not women.

**Fig. 5:**
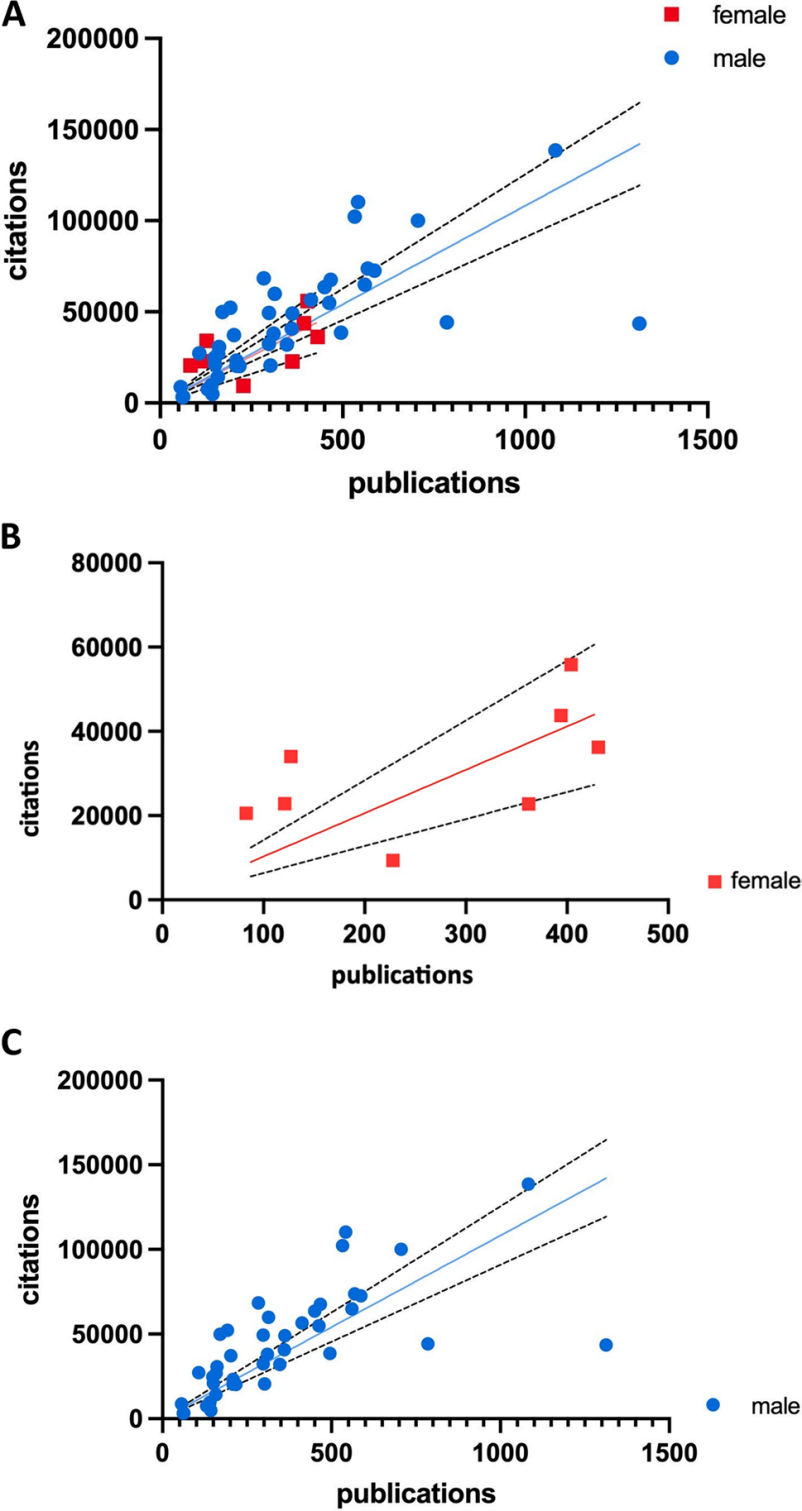
Analysis of the citations in relation to the publications of the Nobel Prize laureates (2006–2022); **A** all laureates, **B** female laureates, **C** male laureates

Figure 5 shows the relation between the number of publications and citations of all Nobel Prize laureates (panel A) and separately for women (panel B) and men (panel C). The point cloud of female Nobel Prize laureates is more scattered than that of male Nobel Prize laureates. Among the male laureates, two laureates stand out as having a significantly higher number of publications and citations than all other laureates. There are no such features among women. The Pearson correlation was calculated to show the correlation between citations and publications. It was *r*=0.763 for women and *r*=0.667 for men. The slope is almost identical for women and men, with a slightly flatter slope for women. Thus, there are no major differences between the genders.

**Fig. 6:**
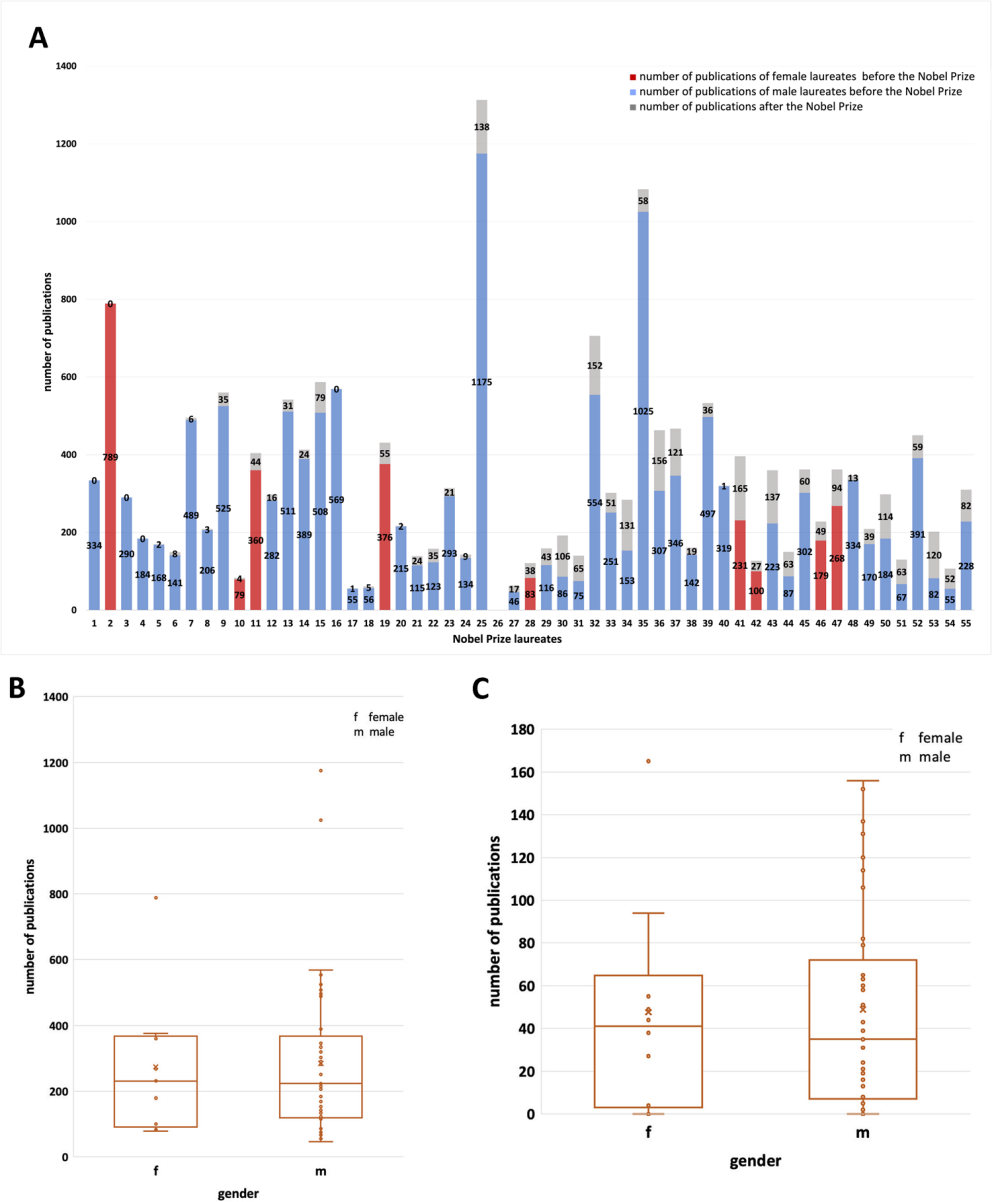
Illustration of publications before the Nobel Prize and publications after the Nobel Prize awarding (Nobel Prize laureates of physiology or medicine and chemistry (in this field only topics related to pharmacology) 2006–2022); **A** publications before and after the Nobel Prize awarding (the numbers 1–55 are representing the order of laureates in Table 1); **B** publications before the Nobel Prize awarding in gender comparison (the *x* is representing the mean value: female 273.889 publications, male 284.489 publications; the dots are representing the number of publications of each Nobel Prize laureate; the box corresponds to the area containing the middle 50% of the data; it is bounded by the upper and lower quartiles; the line centered in the box marks the median values); **C** publications after the Nobel Prize awarding in gender comparison (the *x* is representing the mean value: female 47.6 publications, male 48.82 publications; the dots are representing the number of publications of each Nobel Prize laureate; the box corresponds to the area containing the middle 50% of the data; it is bounded by the upper and lower quartiles; the line centered in the box marks the median values)

Figure 6 shows the individual distribution of publication of Nobel Prize laureates (panel A). Both among men and women, there is a huge variation in the number of publications, ranging from more than 1.200 (Nobel Prize laureate No. 25) to 0 (Nobel Prize laureate No. 26). Overall, most publications of Nobel Prize laureates were published before the award (mean value for women was 273.9; and for men 284.5). After the award, the mean value of publications for women was 47.6, and for men 48.8. This reflects the fact that the award is usually given in late stages of the career (see Fig. 3). However, it should also be noted that most of the researchers are still actively engaged in science after the Nobel Prize award.

**Fig. 7:**
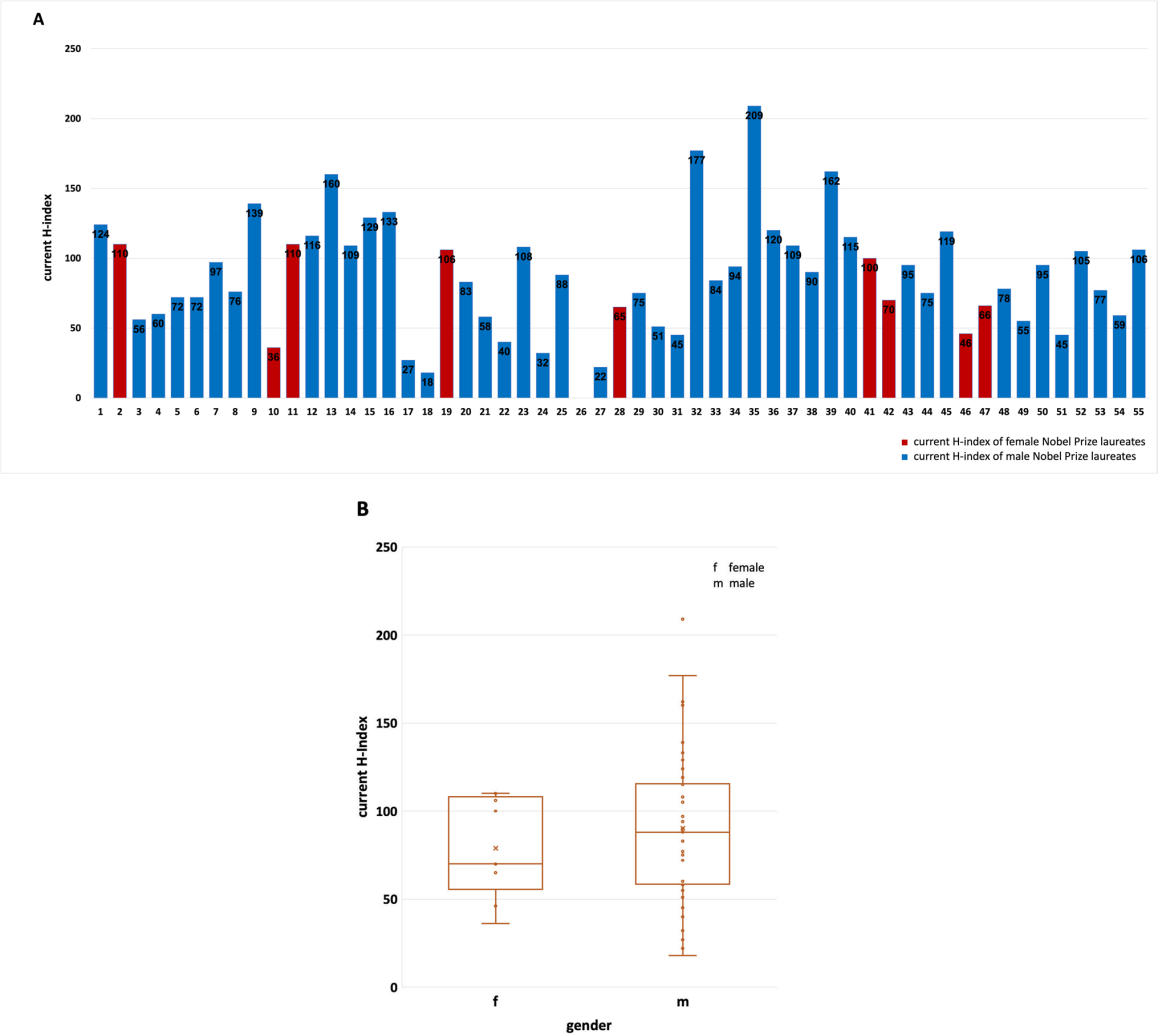
Illustration of the current H-index of Nobel Prize laureates in relation to gender. Nobel Prize laureates of physiology or medicine and chemistry (in this field only topics related to pharmacology) 2006–2022); **A** overview of the current H-index of the laureates (the numbers 1–55 are representing the order of laureates in Table 1); **B** overview of the current H-index of the laureates in relation to gender and SD (the *x* is representing the mean value: female 78.78, male 90.20; the dots are representing the current H-index of each Nobel Prize laureate; the box corresponds to the area containing the middle 50% of the data; it is bounded by the upper and lower quartiles; the line centered in the box marks the median values)

Figure 7 shows the individual H-index (Hirsch-index) distribution among Nobel Prize laureates. Hirsch (2005) defined the H-index as “an index to quantify an individual’s scientific research output. A scientist has index H if H of his or her papers have at least H citations each and the other papers have ≤ H citations each” (Hirsch 2005). The H-index is therefore intended to describe the reception of publications by individual academics in the scientific community.

There is a huge variation in H-index of the Nobel Prize laureates, ranging from > 200 (Nobel Prize laureate No. 35) to 0 (Nobel Prize laureates No. 26). The mean value for women is 78.78, and 90.20 for men (panel B), with men having a much larger variation than women.

**Fig. 8:**
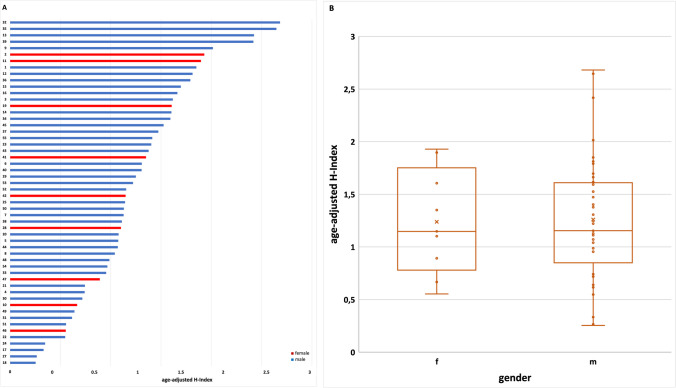
Illustration of the age-adjusted H-index of Nobel Prize laureates. Nobel Prize laureates of physiology or medicine and chemistry (in this field only topics related to pharmacology), 2006–2022; **A** age-adjusted H-Index by listing the numbers of the laureates according to Table 1 (blue, male; red, female); **B** overview of the age-adjusted H-index of the laureates in gender comparison and SD (the *x* is representing the mean value: female 1.24, male 1.26; the dots are representing the current H-index of each Nobel Prize laureate; the box corresponds to the area containing the middle 50% of the data; it is bounded by the upper and lower quartiles; the line centered in the box marks the median values)

The age-adjusted H-index was calculated by dividing the H-index by the age of the Nobel Prize laureates. The results show that women and men do not differ significantly in terms of their age-adjusted H-index (Fig. 8). There was a large variation in this parameter, ranging from > 2.5 (Nobel Prize laureates No. 32 and 35) to 0 (Nobel Prize laureates No. 26). The mean value for women is 1.238, and of men 1.26 with a larger variance by men (0.36) than by women (0.250).

**Fig. 9:**
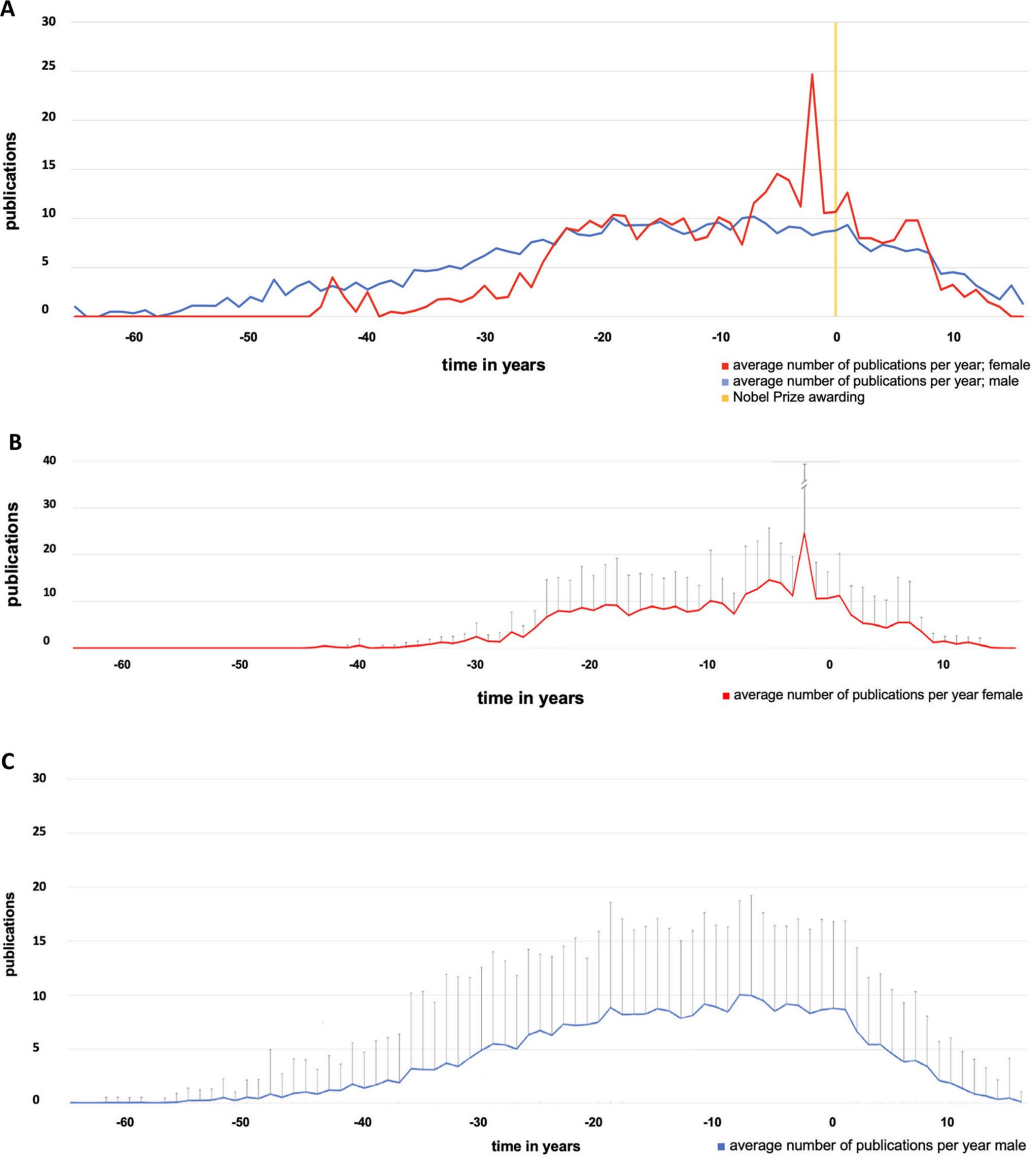
Illustration of the average number of publications of Nobel Prize laureates per year (of the Nobel Prize laureates of physiology or medicine and chemistry (in this field only topics related to pharmacology) 2006–2022); **A** the average number of publications before and after the awarding overall (gender compared), **B** the average number of publications before and after the awarding of female laureates with SD, **C** the average number of publications before and after the awarding of male laureates with SD. The bars in panels **B** and **C** represent the SD

Figure 9 shows the average number of publications per year. The yellow line in panel A shows the year of the Nobel Prize awarding. The years to the left of 0 describe the time before the awarding (with a minus in front of the numbers), the numbers to the right describe the years after the awarding. The number of publications is highest on average at approximately 10 per year for around 20–24 years prior to receiving the Nobel Prize. However, the differences between the individual Nobel Prize laureates are very large. Women and men reach their productivity peak at about the same age. The 20 years immediately before the Nobel Prize awarding (especially the last two years) are more productive for Nobel Prize laureates than the time after the Nobel Prize (Fig. 9).

**Fig. 10:**
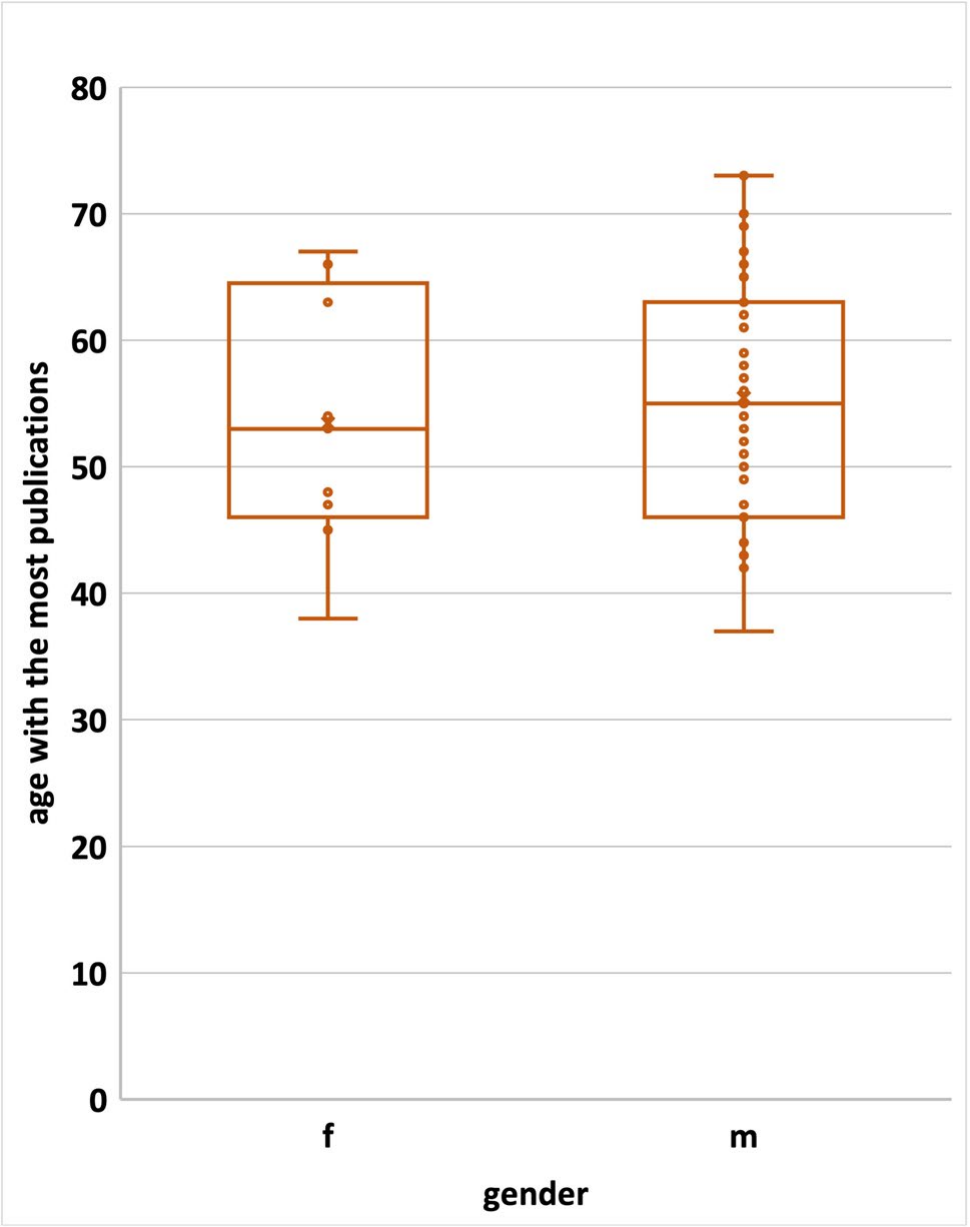
Illustration of the age with the highest productivity (publication peak) of the Nobel Prize laureates of physiology or medicine and chemistry (in this field only topics related to pharmacology) 2006–2022 (the *x* is representing the mean value: female 53.4 years, male 55.44 years; the dots are representing the age in years of each Nobel Prize laureate with the highest productivity; the box corresponds to the area containing the middle 50% of the data; it is bounded by the upper and lower quartiles; the line centered in the box marks the median values)

The average age of the year with the most publications to date is 53.44 years for female Nobel Prize laureates and 55.31 years for male Nobel Prize laureates. The standard deviation is significantly wider for male Nobel Prize laureates than for women (Fig. 10). There was no significant difference between the groups.

**Fig. 11:**
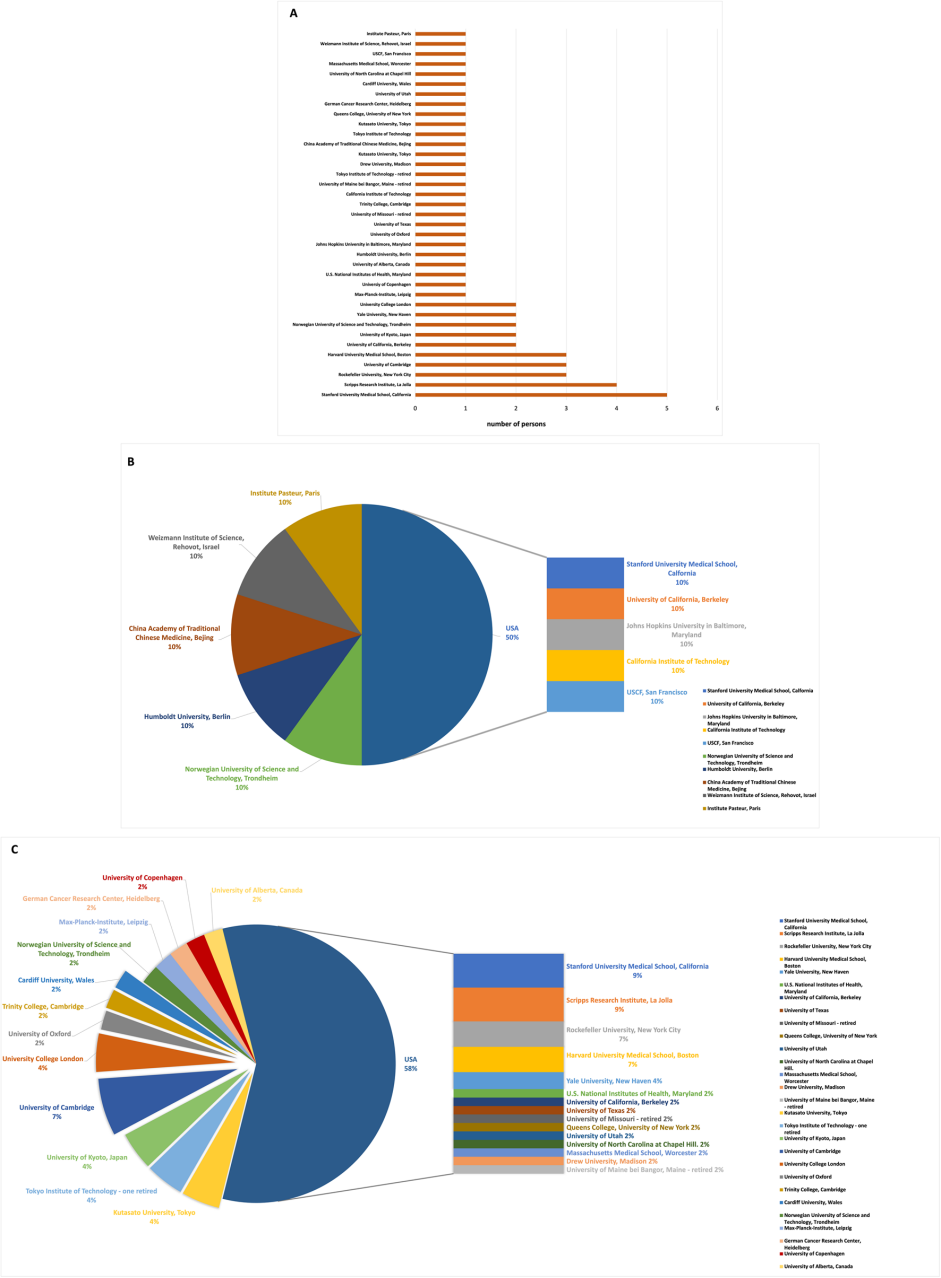
Research locations of Nobel Prize laureates at the time of the Nobel Prize awarding. Nobel Prize laureates of physiology or medicine and chemistry (in this field only topics related to pharmacology), 2006–2022; **A** the research locations by listing the names of the laureates, **B** the research locations of the female laureates, **C** the research locations of the male laureates

Figure 11 shows the research locations at the time of the awarding. The addition of researchers from the University of Stanford, Scripps Institute, Rockefeller University, Harvard University, Yale University, and University of Berkeley (all USA) totals 36% (and therefore more than 1/3), but each individual university is not significantly overrepresented. Most of the other research locations are evenly distributed. Panel B shows the research locations of the female awardees. The 10 female awardees conducted research at 10 different universities, but 50% conducted research at a US university. Among the male awardees (panel C), there is also a fairly balanced distribution of research universities. In a direct comparison of countries, however, 58% of all award laureates conduct their research in the USA, 12% in Japan, 17% in the UK, and just 10% in four other countries.

## Limitations

A limitation of our work is the small database of female Nobel Prize laureates. In addition, we focused on quantifiable bibliometric parameters. Furthermore, there is a very large variation among the individual career paths and productivities of individual Nobel Prize laureates that is not appreciated by our analysis. Most strikingly, even without a single publication and, hence, a non-existant bibliometric track record, important scientific achievements can be made, e.g., 26. We had to limit our bibliometric analysis at a certain calendar date, but it cannot be excluded that in the future, recognition of female scientists having already been awarded the Nobel Prize changes.

Even though the Nobel committees’ mandate is to honor scientific achievements for the benefit of humankind, their interpretation of this criterion was primarily based on their assessment of the groundbreaking nature of the science, while the applied or practical utility of this discovery or bibliometric values such as number of publications, citations, or H-index assessed in the current study are at best secondary factors when awarding the prize (Källstrand 2022). In fact, some Nobel Prize laureates (e.g., 17, 18, 23, 26, 40) have only few publications or no publications. Hansson et al. (2019) state that it is difficult to measure this “greatest benefit to mankind” or brilliance in science in an objective way.

## Conclusions

To the best of our knowledge, this is the first study that aims at providing a bibliometric comparison of female and male Nobel Prize laureates. Based on numerous studies pointing to a discrimination of women in science (Ceci and Williams 2011; Moss-Racusin et al. 2012; Ball 2023; Beaudry and Larivière 2016; Ceci and Williams 2007; Charyton et al. 2011; Harding 1998; Kulis and Sicotte 2002; Lubinski et al. 2001; Ma et al. 2019; Ross et al. 2022), it cannot be excluded that even among this group of absolute elite scientists, some sort of discrimination occurs. However, looking on numerous bibliometric parameters, we did not obtain evidence for a bias against women. Rather, for crucial parameters such as publications before the Nobel Prize, citations, age-adjusted H-index, productivity peak, and research location, we did not find evidence for systematic discrimination of female Nobel Prize laureates relative to male Nobel Prize laureates. Rather, women were awarded the Nobel Prize at a significantly younger age than men although both genders have a similar age with regard to the peak of research productivity. Thus, surprisingly, our study shows that the research accomplishments of female Nobel Prize laureates are actually recognized earlier than those of men. This strongly argues against the Nobel Prize committee being discriminatory against women although the current Nobel assembly is male-dominated.

There are six Nobel Committee members for physiology or medicine, five male members and just one female member (https://www.nobelprize.org/about/the-nobel-committee-for-physiology-or-medicine/; last accessed 03/29/2024). In case of systematic discrimination of females, we would have expected that female Nobel Prize laureates are much older than their male counterparts and need to have many more publications and citations and a higher H-index. This was, however, not the case. We also did not notice overrepresentation of a specific country or research institution among female Nobel Prize laureates. Thus, it appears that the current Nobel Committee tries to look for the best candidates for the Nobel Prize independently of gender. This is supported by the fact that concerning contemporary Nobel Prize laureates in the topics discussed here (Table 1), there has never been such an egregious case of omitting females as the non-consideration of Rosalind Franklin who made seminal contributions to the identification of the DNA structure (Conti 2021).

The most controversial case of non-consideration for the Nobel Prize in recent times in the fields considered here probably concerns a male (Salvador Moncada for the nitric oxide/cGMP pathway), where bias against him coming from a developing country was speculated to have played a role (Lancaster 1998). In the present study, representation of citizens from developing countries is poor as well (Table 1). Scientists coming from developed countries dominate the field regarding Nobel Prize awards.

The number of female Nobel Prize laureates with a relation to pharmacology is much smaller than the number of male Nobel Prize laureates. A gender gap is not only observed for the Nobel Prize but also for other scientific awards (Hansson 2023). Hence, our present study complements the current knowledge on gender imbalance concerning scientific awards.

The study of Zehetbauer et al. (2022) showed that the number of female first authors in pharmacology-related papers, mostly reflecting PhD students and postdocs, is much higher than the number of female senior authors, the latter reflecting group leaders conducting independent research. This study suggests that the major drop of female researchers occurs between the PhD student and postdoc stage versus group leader stage. This career stage often collides with family planning. Thus, a major factor accounting for the small number of female Nobel Prize laureates is the smaller number of female researchers who enter an intellectually independent research career: an unwritten prerequisite for getting eligible for the Nobel Prize. All of the Nobel Prize laureates in Table 1 fulfill the criterion of long-term research as intellectually independent investigator.

But it must also be taken into consideration that both female and male scientists are not just passive objects in a career system but that they also make active decisions about what they do and what they do not do in their scientific careers (Zöllner and Seifert 2024). The latter study epitomized that female German pharmacologists invest much less in social capital (scientific visibility in the German science community via the journal “Biospektrum”) than their male counterparts although they are very much encouraged to do so by the Executive Board of the German Pharmacological Society and although the time effort needed to become visible is low. Visibilty is important for being recognized a potential award candidate. The study also noted substantial gender differences between various scientific fields regarding investment in visibility. The aspect of voluntary conscious decisions of individuals is, unfortunately, substantially underrated in the current gender discussion in science.

## Future studies

The group of Nobel Prize laureates is a very small group of elite researchers, and only the minority of all important research accomplishments is awarded the Nobel Prize (Pohar and Hansson 2020). Thus, it will be very important to expand this type of bibliometric research to a larger population of scientists, independently of an award. One approach could be to analyze, the group of the leading 10.000 or 100.000 scientists globally and relying on an integrative approach including number of publications, citations, and H-index. The advantage of analyzing many scientists is that it is much easier to analyze cultural differences among different countries. It will also be worthwhile, in 10 years from now, to repeat the current study and compare how Nobel Prize laureates from 2006 to 2022 compare with Nobel Prize laureates from 2023 to 2032. Interviews should be conducted with scientists regarding their professional choices. Lastly, it will be important to analyze the contributions of scientists from developing countries, both male and female, who may not have received the Nobel Prize.

## Data Availability

All source data for this study are available upon reasonable request.

## Abbreviations

ANOVA: Analysis of variance
*f*: Female
H-index: Hirsch index
m: Male
MCA: Multiple correspondence analysis
MW: Mean value
NP: Nobel Prize
QS: Quacquarelli Symonds/World University Ranking
SD: Standard deviation
USA: United States of America
